# Supplementary material for: Impacts of Side Effects to BNT162b2 and the First Dose of ChAdOx1 Anti-SARS-CoV-2 Vaccination on Work Productivity, the Need for Medical Attention, and Vaccine Acceptance: A Multicenter Survey on Healthcare Workers in Referral Teaching Hospitals in the Republic of Korea
Source: Vaccines (Basel). 2021 Jun 14;9(6):648. doi: 10.3390/vaccines9060648 (PMC8232011; doi:10.3390/vaccines9060648)
Supplement: Supplementary file 1 [file vaccines-09-00648-s001.zip › vaccines-1235304-supplementary.pdf]

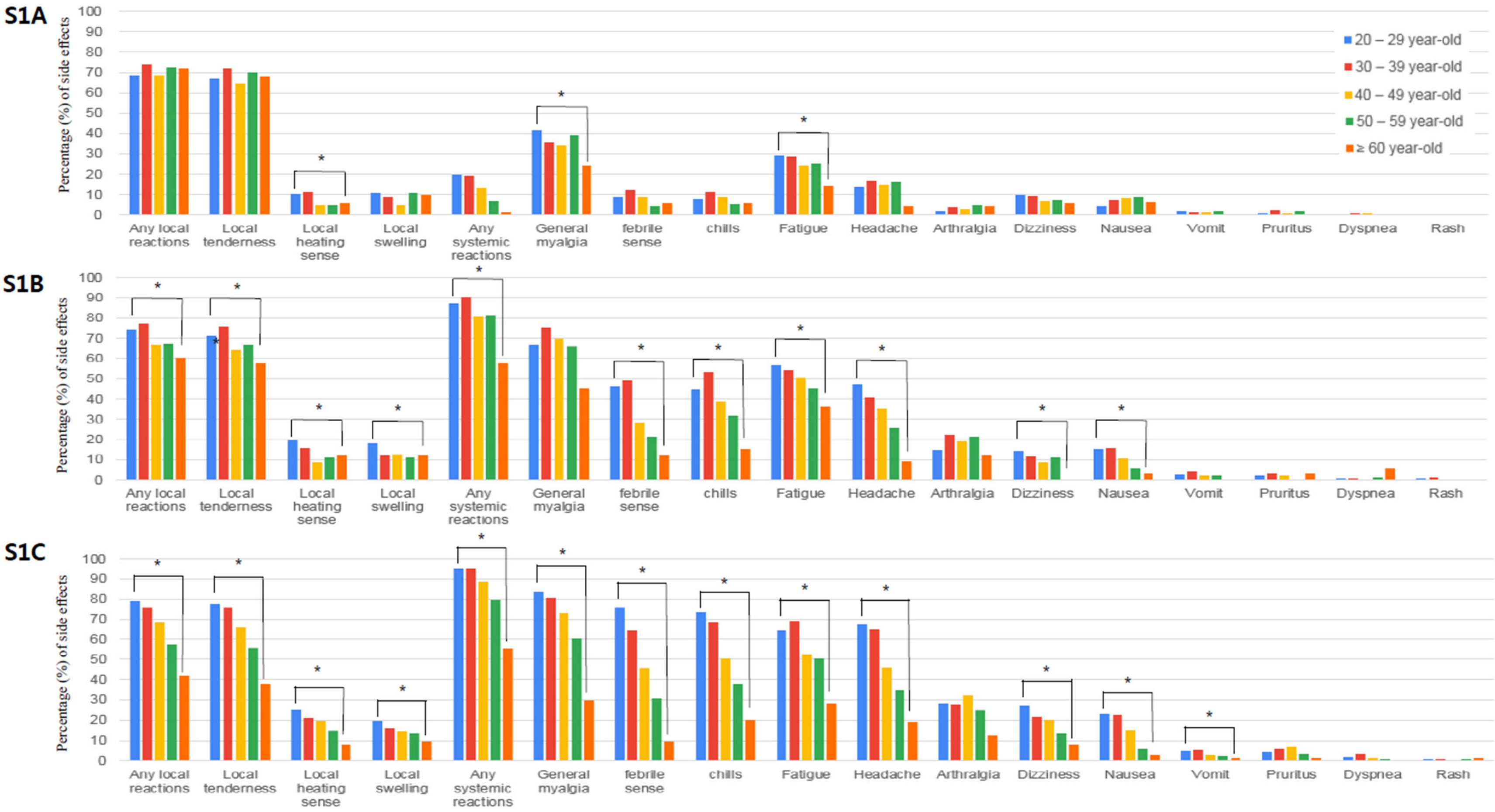

Figure S1. Adverse reactions after anti-SARS-CoV-2 vaccination according to age groups. Figure S1A, adverse reactions in theChAdOx1 #1 group. Figure S1B, adverse reactions inthe BNT162b2 #1 group. Figure S1C, adverse reactions in the BNT162b2 #2 group. ChAdOx1 #1, the first dose of ChAdOx1; BNT162b2 #1, the first dose of BNT162b2; BNT162b2 #2, the second dose of BNT162b2.*, trend of a signicant decrease in the frequency of adverse reactions across age groups.

Table S1. Side effects and their impacts on the intention to receive the second dose of ChAdOx1 vaccine on various age groups.

**Intention to receive the second dose**	**20–29 year-old**			**30–39 year-old**			**40–49 year-old**			**50–59 year-old**		
	**Yes (*n* = 470)**	**No (*n* = 143)**	***p*-value**	**Yes (*n* = 268)**	**No (*n* = 99)**	***p*-value**	**Yes (*n* = 286)**	**No (*n* = 74)**	***p*-value**	**Yes (*n* = 228)**	**No (*n* = 37)**	***p*-value**
Local reactions	361 (76.8)	124 (86.7)	0.01	197 (73.5)	82 (82.8)	0.07	190 (66.4)	57 (77.0)	0.09	130 (57.0)	23 (62.2)	0.60
Local tenderness	355 (75.5)	121 (84.6)	0.02	196 (73.1)	82 (82.8)	0.06	184 (64.3)	55 (74.3)	0.13	125 (54.6)	22 (59.5)	0.72
Local erythema/heating sense	105 (22.3)	52 (36.4)	<0.01	47 (17.5)	30 (30.3)	0.01	42 (14.7)	28 (37.8)	<0.01	30 (13.2)	9 (24.3)	0.08
Local edema	77 (16.4)	43 (30.1)	<0.01	34 (12.7)	26 (26.3)	<0.01	33 (11.5)	18 (24.3)	0.01	29 (12.7)	6 (16.2)	0.60
Systemic reactions	443 (94.3)	139 (97.2)	0.19	252 (94.0)	96 (97.0)	0.42	247 (86.3)	72 (97.3)	0.01	180 (78.9)	31 (83.8)	1.00
General myalgia	383 (81.5)	129 (90.2)	0.01	209 (78.0)	87 (87.9)	0.04	203 (71.0)	59 (79.7)	0.15	133 (58.3)	27 (73.0)	0.11
Febrile sense	352 (74.9)	113 (79.0)	0.37	165 (61.6)	71 (71.7)	0.09	121 (42.3)	43 (58.1)	0.02	67 (28.4)	15 (40.5)	0.18
Chills	332 (70.6)	117 (81.8)	0.01	175 (65.3)	76 (76.8)	0.04	136 (47.6)	46 (62.2)	0.03	78 (34.2)	22 (59.5)	0.01
Fatigue	283 (60.2)	110 (76.9)	<0.01	177 (66.0)	77 (77.8)	0.03	142 (49.7)	47 (63.5)	0.04	114 (50.0)	19 (51.4)	1.00
Headache	297 (63.2)	117 (81.8)	<0.01	161 (60.1)	77 (77.8)	<0.01	118 (41.3)	48 (64.9)	<0.01	70 (30.7)	22 (59.5)	<0.01
Arthralgia	125 (26.6)	47 (32.9)	0.17	69 (25.7)	33 (33.3)	0.15	88 (30.8)	27 (36.5)	0.40	53 (23.2)	13 (35.1)	0.15
Dizziness	120 (25.5)	48 (33.6)	0.07	47 (17.5)	31 (31.3)	0.01	55 (19.2)	18 (24.3)	0.33	27 (11.8)	9 (24.3)	0.07
Nausea	104 (22.1)	37 (25.8)	0.37	61 (22.8)	22 (22.2)	1.00	37 (12.9)	18 (24.3)	0.02	13 (5.7)	4 (10.8)	0.27
Vomit	22 (4.7)	10 (7.0)	0.29	12 (4.5)	9 (9.1)	0.13	6 (2.1)	4 (5.4)	0.13	3 (1.3)	3 (8.1)	0.04
Pruritus	120 (25.5)	48 (33.6)	0.07	15 (5.6)	8 (8.1)	0.47	19 (6.6)	6 (8.1)	0.61	7 (3.1)	2 (5.4)	0.37
Dyspnea	5 (1.1)	7 (4.9)	0.01	7 (2.8)	5 (5.1)	0.32	2 (0.7)	3 (4.1)	0.06	1 (0.4)	2 (5.4)	0.05
Rash	2 (0.4)	4 (2.8)	0.03	2 (0.7)	2 (2.0)	0.30	0	1 (1.4)	0.21	0	3 (8.1)	0.01

Data are presented as numbers (%) of respondents. Answers of respondents aged ≥ 60 year-old group were not analyzed, because only three (4.1%) respondents refused to receive the second dose of ChAdOx1.
